# Aspects of Histopathological and Ultrastructural Retinal Changes in Chronic Exposure to Hydroxychloroquine

**DOI:** 10.3390/medicina60060846

**Published:** 2024-05-22

**Authors:** Aida Geamănu, Ancuţa Elena Baciu, Ruxandra Pirvulescu, Raluca Iancu, Nicoleta Anton, Alina Popa-Cherecheanu, Aurelian Mihai Ghita, Mihaela Oana Romanitan

**Affiliations:** 1 Department of Ophthalmology, “Carol Davila” University of Medicine and Pharmacy, 020021 Bucharest, Romania; aida.geamanu@umfcd.ro (A.G.); alina.cherecheanu@umfcd.ro (A.P.-C.); 2 Department of Radiotherapy, Institute of Oncology Prof. Dr. “Alexandru Trestioreanu”, 022328 Bucharest, Romania; ancutabaciu@gmail.com; 3 Department of Ophthalmology, “Grigore T. Popa” University of Medicine and Pharmacy, 700115 Iasi, Romania; nicolofta@gmail.com; 4 Department of Physiology, “Carol Davila” University of Medicine and Pharmacy, 020021 Bucharest, Romania; ghita.amg@gmail.com; 5 Department of Internal Medicine, Section of Neurology, Södersjukhuset, 11883 Stockholm, Sweden; mihaela.romanita@regionstockholm.se

**Keywords:** hydroxychloroquine, rat, retinal toxicity, histopathology

## Abstract

*Background and Objective:* Hydroxychloroquine sulfate (HCQ) is a lysosomotropic agent administered in systemic lupus erythematosus and rheumatoid arthritis that has fewer toxic effects than chloroquine. However, HCQ may still be responsible for retinal toxicity. In this study, we observed structural changes in the retinas of experimental rats after prolonged exposure to HCQ. *Matherials and Methods:* We investigated several aspects regarding retinal changes, at both the histopathological and ultrastructural levels. We used 96 male albino Wistar rats distributed into four equal groups (n = 24 per group): the first three groups were treated with different doses of HCQ (50, 100, and 200 mg/kg HCQ, injected intraperitoneally in a single dose daily), and the last group (the control group, n = 24) was treated with saline solution administered in the same way (0.4 mL of saline solution). The treated groups received HCQ daily for 4 months, and every month, six animals from each group were sacrificed to assess retinal changes. The eyes were examined via optical (OM) and electronic microscopy (EM). Statistical analysis was deployed, and results regarding retinal morpho-photometry were acquired. *Results:* We observed structural retinal changes in both high and low doses of HCQ; while high doses determined a significant thinning of the retina, lower doses caused retinal thickening. Morphological retinal changes upon exposure to HCQ are believed to be caused by accumulated HCQ in lysosomes found in retinal ganglion cells and in the inner nuclear and photoreceptor cell layers. Such changes were most evident in the group receiving HCQ intraperitoneally in doses of 100 mg/kg for a longer period (4 months). *Conclusions:* The present study highlights histopathological and ultrastructural retinal changes induced by chronic HCQ administration, which were strongly connected to the dosage and period of exposure.

## 1. Introduction

Hydroxychloroquine sulfate (HCQ) belongs to a group of medicines known as antimalarials; this type of substance modulates the immune response and acts as a lysosomotropic agent. It is administered in the treatment of systemic lupus erythematosus rheumatoid arthritis and other autoimmune diseases, such as dermatological diseases, and has much fewer toxic effects than chloroquine [1]. Toxicity occurs rarely, but it is potentially severe and involves various combinations of retinal injuries with associated neuromuscular, cardiac, hematologic, and gastrointestinal deficits. The hydroxyl group added to the chloroquine formula to form the HCQ agent limits access to the blood–retina barrier and thus reduces ocular toxicity [2,3].

HCQ-induced retinal toxicity has been reproduced in several animal species, including rats, cats, dogs, rabbits, pigs, and monkeys. An interesting hypothesis was recently proposed stating that HCQ could even have the effect of preventing age-related macular degeneration (ARMD) in subjects with rheumatoid arthritis [4]. ARMD is the most common cause of blindness in the elderly population, and local chronic inflammation plays a role in the pathogenesis and progression of this disease [5].

The mechanism of retinal toxicity induced by both chloroquine and hydroxychloroquine is not fully explained. It is supposed that the primary lesions occur in the retinal pigment epithelium (RPE) and photoreceptors. Previous studies on mice suggest that photoreceptors undergo reversible inner retinal lipidosis as well as irreversible and progressive degeneration, despite ceasing medication [6].

Hallberg et al. studied retinal effects of long-term chloroquine exposure in mice; it appears that primarily, there are effects on phospholipid metabolism that are noticed in retinal ganglion cells (RGCs), but not in the photoreceptors or RPE. This study suggests that RGCs may be initially or primarily affected by CQ exposure, which induced the hypothesis that ganglion cell structure, as well as their axons and retinal nervous fiber layer (RNFL), could be affected in patients who have been taking CQ for a long time [7].

Epidemiological studies have shown that chronic rheumatoid arthritis patients treated with anti-inflammatory agents have a lower risk of developing degenerative diseases such as Alzheimer’s or Parkinson’s [8,9]. A study published in 2005 developed by McGeer and Sibley showed a lower incidence of degenerative maculopathies in rheumatoid arthritis subjects treated with anti-inflammatory medication for a long period, especially those treated with HCQ [10].

This study aims to highlight retinal histopathological changes induced by chronic exposure to hydroxychloroquine in the early stages in experimental animals (rats). To achieve this objective, 96 male Wistar rats were treated with HCQ in different doses, and a control group was treated with saline solution. Retinal analysis was performed using two microscopic methods (optical and electronic microscopy).

## 2. Materials and Methods

The experimental rats were provided and hosted by the “Victor Babes” National Research and Development Institute for Pathology and Biomedical Sciences. 

All experiments and animal research were conducted according to the Declaration of Helsinki. This study received the approval of the Ethics Committee of Scientific Research of the “Carol Davila” University of Medicine and Pharmacy, Bucharest, Romania, Issue No. 84/02.03.2021.

The animals were divided into 4 groups of 6. The ambient temperature was 22 ± 2 °C, and humidity was 50–60%. Artificial lighting was provided while alternating 12 h of light/12 h of no light. All the animals received the same diet (which was a proper rodent diet recommended by a veterinarian—root vegetables).

HCQ (200 mg tablets procured from Sanofi Company (Paris, France)) was dissolved in saline solution.

Three groups were treated with different doses of HCQ (50, 100, and 200 mg/kg of HCQ injected intraperitoneally, administered as single dose daily), and one control group—control group M (n = 24)—was administered saline solution in the same way, all in a volume of 0.4 mL. Group A received doses of 50 mg/kg for 1 month (subgroup 1 A), 2 months (subgroup 2 A), 3 months (subgroup 3 A), and 4 months (subgroup 4 A). Group B received 100 mg/kg HCQ for 1 month (subgroup 1 B), 2 months (subgroup 2 B), 3 months (subgroup 3 B), and 4 months (subgroup 4 B) (see Table 1). In the third group, which received 200 mg/kg of HCQ, within 48 h of the beginning of our study, all rats were found to have spontaneously died, so we decided to abandon this batch considering the high toxicity risk of the dose. The other two treated groups (groups A and B) (n = 48) received HCQ over 4 months (in dosages of 50 and 100 mg/kg). Each month, the 6 animals from the respective subgroups (1, 2, 3, 4) of all 3 remaining groups (treatment groups A and B and control group M) were intentionally sacrificed as part of our experiment, and retinal changes were analyzed. Inhalator anesthesia with Chloroform was used; the protocol for all this procedure is presented in Table 2.

The eyes were collected, processed, and dissected for optical microscopy and electronic microscopy.

### 2.1. Preparation of Retinal Tissue for Optical Microscopy (OM)

The tissues were fixed in 10% formaldehyde and kept for 24 h at room temperature. Following this procedure, the prepared samples were washed with water and later dehydrated with alcohol. After dehydration, we started the process of clarifying the samples in isopropyl alcohol. The next stage involved immersing the parts in paraffin, resulting in paraffin blocks that were placed in a freezer. Sections of 2 μm thickness were cut, followed by mounting on glass slides. The glass slides were placed in a thermostat for 24 h after being stained.

The staining technique included several operations. The slides were dewaxed using toluene for 30 min to 1 h, clarified in ethanol, washed in tap water, stained with Mayer’s hematoxylin for 10 min, and then thoroughly washed again in tap water. The next step in the staining process involved highlighting the nuclei using lithium carbonate for 10 s, after which the samples were washed and stained with alcoholic eosin for 5 min and then washed again. Excess dye was removed using three ethanol baths (70%, 96%, and absolute ethanol). Sections stained with hematoxylin and eosin (HE) were analyzed via OM imaging using a Nikon SMZ 10 (ORIGINALMIND, Okaya, Nagano, Japan).

### 2.2. Preparation of Retinal Tissue for Electronic Microscopy (EM)

For EM, as soon as the ocular globes were harvested, they were fixed by immersion in 4% buffered glutaraldehyde in sodium cacodylate 1 M (Agar Scientific, Essex, UK). After 1 min, the globes were fragmented into small pieces of 1 mm^3^ for better sample fixation. The retinal samples were fixed for 24 h, postfixed with osmium tetroxide 1%, and prepared for inclusion of epoxy resin (Agar 100, Agar Scientific UK). Semithin sections were cut with a Leica Ultramicrotome (Leica Microsystems, Wetzlar, Germany) and stained with toluene blue (Agar Scientific) for structural analysis. Ultrathin sections (80–100 nm) were cut with a Leica Ultramicrotome (Leica Microsystems, Germany) and stained on grids with uranyl acetate and Reynolds’s lead citrate solutions (Agar Scientific, UK), and were then examined using a Morgagni 286 TEM (FEI Company, Eindhoven, The Nederlands) at 80 kV. Digital electron micrographs were recorded with a MegaView III CCD using iTEM-SIS software (Olympus Soft Imaging System, Germany).

### 2.3. Statistics and Morphometric Analysis

HE-stained sections were analyzed using the iTEM-SIS imaging software (Olympus Soft Imaging System GmbH, Münster, Germany). Information was obtained regarding the total retinal thickness, internal retinal layer thickness (measured from the internal limiting membrane to the inner nuclear layer), outer retinal layer thickness (outer plexiform layer to the retinal pigment epithelium layer), and the thickness of the retinal pigment epithelium. Data from the experimental groups were analyzed statistically and compared with the control group data using the IBM SPSS program (29.0, Armonk, NY, USA).

## 3. Results

### 3.1. Pathological Examination

**Figure 1:**
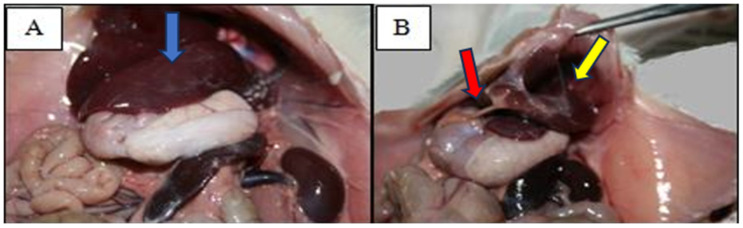
Pathological examination of one deceased rat in group C. The animal was intraperitoneally administered a single dose of 200 mg/kg of HCQ. Hepatosplenomegaly ((**A**), blue arrow) can be observed, as well as adhesions ((**B**), red arrow) and white deposits ((**B**), yellow arrow) on the abdominal organs and a serous-looking splashed white abdomen.

In the deceased animal group (group C), pathological examination provided the following observations: hepatomegaly and splenomegaly, significant intra-abdominal adhesions, and whitish deposits scattered unevenly throughout all serous abdominal organs (Figure 1A,B). This particular and detailed pathological exam was performed only for group C, in which all animals died spontaneously within 48 h of the beginning of our experiment. The cause of death was determined to be multi-system organ failure caused by an HCQ overdose (200 mg/kg). The other two groups of experimental animals (group A and group B) did not undergo the same pathological exam; these two groups were sacrificed intentionally at the previously specified intervals (six animals per month per subgroup), and histopathological examinations were focused only on the ocular segment.

### 3.2. Retinal Microtopography

After analyzing the retinal surface microtopography, we measured the average values of all the retinal layers. These values can be viewed in Table 3.

The values in Table 3 reveal subtle thickening in the retinas in all A subgroups and significant retinal thinning in all B subgroups; compared with the control group, these modifications were more obvious and statistically significant after 4 months (in subgroup 4 B) than at all earlier time points.

Animals from group A that received a lower dose of HCQ (50 mg/kg) presented a thickened retina in both the inner and outer layers; this thickening was statistically significant in the outer retinal layer in comparison with the control group and was more obvious after the second month of drug exposure.

In subgroup 4 B (the group with continuous daily administration of HCQ 100 mg/kg for four months), marked thinning in total retinal thickness was observed, a thinning of 36 μm compared with the control group, thus explaining the toxic effect of HCQ on retinal structures. Our hypothesis is related to a study published in 2022 by Melles et al.; the purpose of the study was to highlight the relationship between retinal thinning and HCQ retinopathy in patients with chronic HCQ intake. While retinal thickness remains stable for many years in most patients receiving long-term hydroxychloroquine therapy, there is a turning point when the retina may begin to thin rapidly. The study provided objective evidence that these early structural changes may occur several years before conventional signs of HCQ retinopathy appear [11].

In subgroup 4 A, there was a statistically significant thickening of the total retina, by 11 μm, compared with the controls. In subgroup 4 B, we observed marked retinal thinning compared with the control group, with an inner retinal thickness of 32 μm. Outer retina thinning by approximately 4 μm was observed in subgroup 4 B compared with the control group. These findings indicate the strong cumulative toxic effect of HCQ on retinal structures, generating irreversible, destructive lesions; both the thickening in group A and the thinning in group B continuously increased over time from 1 A to 4 A and from 1 B to 4 B.

In group A (with 50 mg/kg) we noticed an increase in the RPE thickness, more noticeable at 4 months from HCQ exposure. In group B (with 100 mg/kg of HCQ), we noticed a decrease in RPE thickness, which was more evident after 4 months of HCQ exposure. The thickening of the RPE was more obvious during the last month of drug exposure in subgroup 4 A (4.17 μm) compared with the control group (see Table 3). This RPE thickening could be attributed to an eosinophilic substance generically called hyaline (this substance has a variable chemical structure, does not induce inflammation, and is well tolerated). This fact could be explained by hyaline acting as a possible mechanical barrier against the penetration of neo-vessels produced by inflammatory processes present in various retinal diseases [12].

### 3.3. Structural Analysis of the Retina Using Optical Microscopy

**Figure 2:**
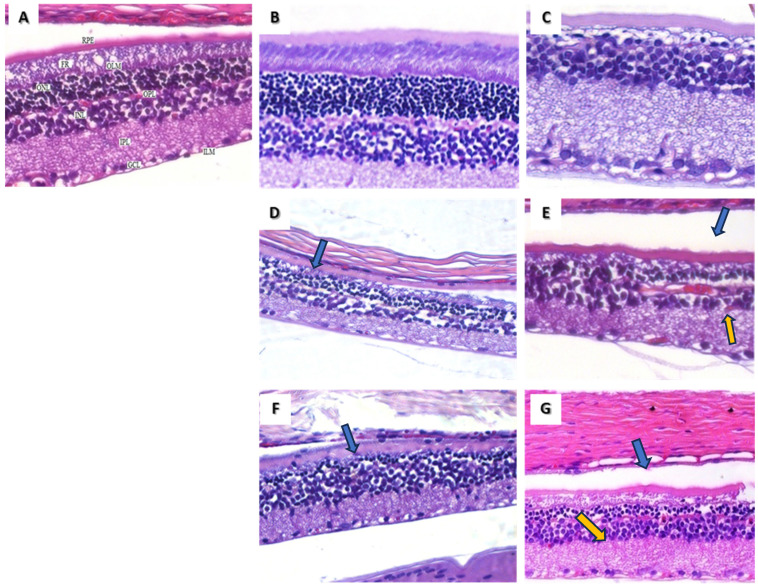
Light microscopy images of rat retina sections. (**A**) Retina from the control group—(RPE = retinal pigment epithelium, FR = retinal fiber layer, OLM = outer limiting membrane, ONL = outer nuclear layer, OPL = outer plexiform layer, INL = inner nuclear layer, IPL = inner plexiform layer, GCL = ganglion cell layer, and ILM = inner limiting membrane). (**B**)—subgroup 1 A, showing apparently normal retinal layers. (**C**)—subgroup 1 B, showing apparently normal retinal layers. (**D**)—subgroup 2 B, presenting thinning areas localized at the outer retinal plexiform layer and damage at the photoreceptor layer level (blue arrow). (**E**)—subgroup 3 A shows thickening in the inner retinal layers, especially the IPL (yellow arrow) and RPE (blue arrow), compared with the control group. (**F**)—subgroup 3 B shows thinning in the outer plexiform retinal layer and a fusion between areas of the outer and inner retinal layer (blue arrow). Notice the low number of ganglion cells in this stage. (**G**)—subgroup 4 A: notice the normal cellular architecture and thickening in the IPL (yellow arrow), with no changes in the nuclear layers, with thicker focal areas of the RPE (blue arrow) compared to the control group. H&E stain magnification 400×.

Structural changes in the retina caused by chronic exposure to HCQ were highlighted using OM. The retinal samples in the control group were analyzed from outside to inside: retinal pigment epithelium (RPE), photoreceptor cell layer (FR), outer limiting membrane (OLM), outer nuclear layer (ONL), outer plexiform layer (OPL), inner nuclear layer (INL), inner plexiform layer (IPL), ganglion cell layer (GCL), retinal nerve fiber layer (RNFL), and inner limiting membrane (ILM) (Figure 2A).

The retinas of rats injected with 50 mg/kg and 100 mg/kg of HCQ for a month (subgroups 1 A and 1 B) revealed no structural changes compared with the control group (Figure 2B,C). However, at the end of the second month of HCQ exposure (subgroup 2 B), a few focal areas of thinning in the outer plexiform layer were observed (Figure 2D).

After three months of low-dose HCQ administration (50 mg/kg; subgroup 3 A), a histopathological examination showed visible thickening in the inner retina (from the internal limiting membrane to the internal nuclear layer) and a visible thickening of the outer retina. In addition, there was an increase in retinal pigment epithelial thickness relative to the control group (Figure 2E).

In contrast to that, in rats injected with the higher dose of HCQ for three months (subgroup 3 B), we observed an outer retinal layer thinning with a fusion between the outer nuclear layer and internal nuclear layer in certain areas. We also noticed a visible loss of retinal ganglion cells (Figure 2F). Four months after starting the present study, in subgroup 4 A, we observed an apparent thickening in the internal plexiform layer, without changes in the retinal nuclear layer; we also observed slightly thickened areas on parts of the retinal pigment epithelium (Figure 2G).

In the last month of our experiment, we noticed that the retinal architecture was preserved, and layers could be easily distinguished at HCQ doses of 50 mg/kg. However, at higher doses of HCQ (100 mg/kg), the following was observed: cellular anarchy, starting with the outer plexiform retinal layers; loss in some areas, with nuclear cell fusion, thinning in the inner plexiform layer and inner nuclear layer; and massive edema with significant damage to the ganglion cell layer (Figure 2D,F).

Optical microscopy and an assessment of the retina—morpho-photometry—showed thickening in the retinal pigment epithelium in the low-dose group (Figure 2G), which can be explained by the body creating a possible mechanical barrier against neo-vessel penetration caused by the activation of inflammatory processes produced in various retinal disorders [12].

### 3.4. Structural Assessment of the Retina with Electronic Microscopy

**Figure 3:**
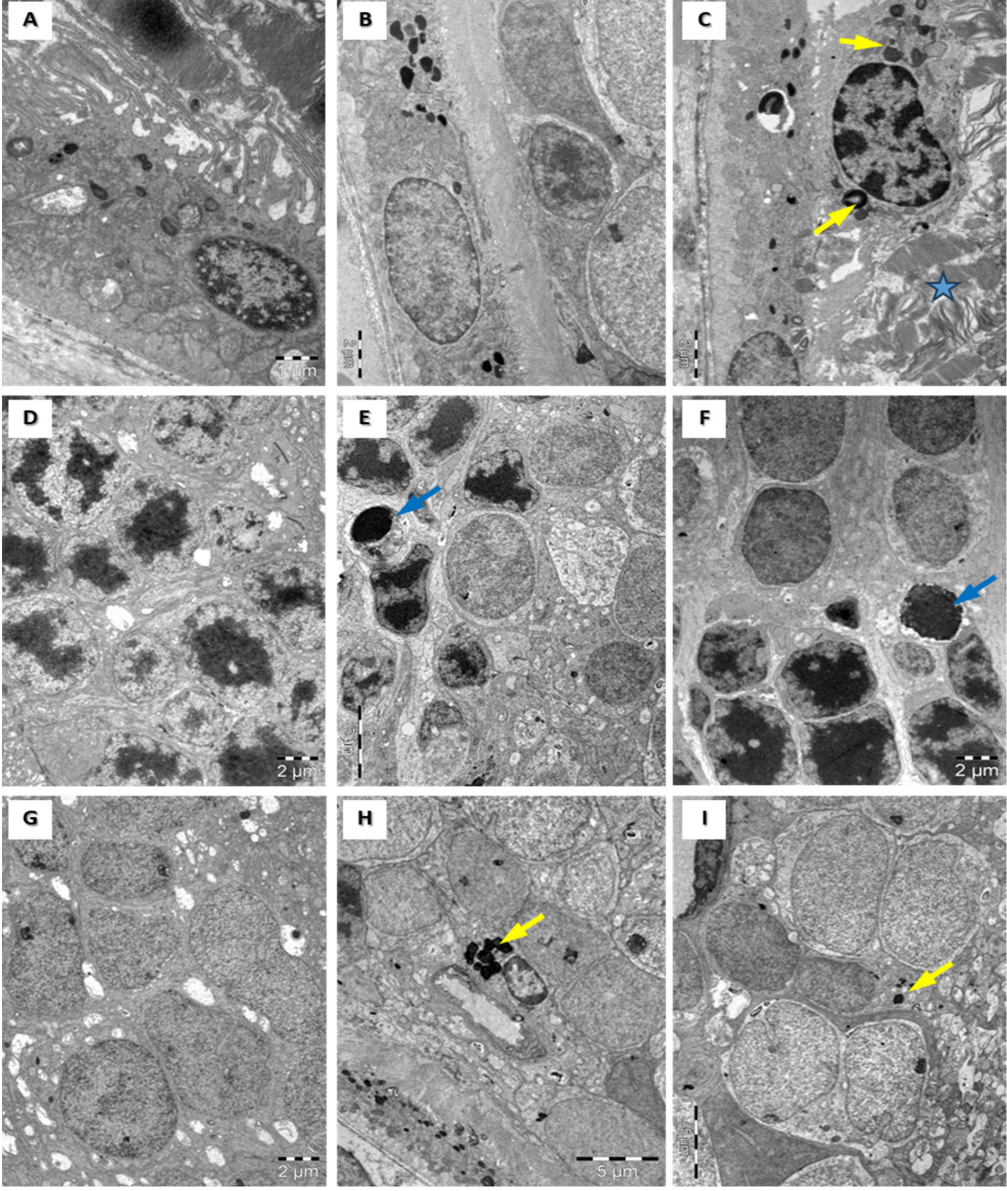
Transmission electron microscopy images of rat retinas from control (**A**,**D**,**G**), subgroup 3 A (**B**,**E**,**H**), and subgroup 2 B (**C**,**F**,**I**) show that retinal lesions depend on dosage—the higher the dose, the earlier the lesions. External segments of rod and cone cells and retinal pigment cells in the outer retina (**A**–**C**). The outer nuclear layer (**D**–**F**). The ganglion cell layer (**G**–**I**). The outer retina shows normal ultrastructure in control (**A**) and subgroup 3 A (**B**). Highly altered rod and cone ultrastructure (blue star) and macrophages with lysosomes (yellow arrows) are visible in subgroup 2 B (**C**). Apoptosis (blue arrows) is visible in the outer nuclear layer in subgroups 3 A (**E**) and 2 B (**F**). An increased number of lysosomes in the ganglion cell layer cells is visible in subgroups 3 A (**H**) and 2 B (**I**). Scale bars: 1 µm (**A**), 2 µm (**B**–**D**,**F**), and 5 µm (**E**,**H**,**I**).

An ultrastructural examination of retinas in the control group showed cell nuclei, highlighted retinal pigmentation, the external segment of the photoreceptor layer (Figure 3A), and the internal segment of the photoreceptor layer (Figure 3D), as well as the ganglion cell layer (GCL) (Figure 3G).

**Figure 4:**
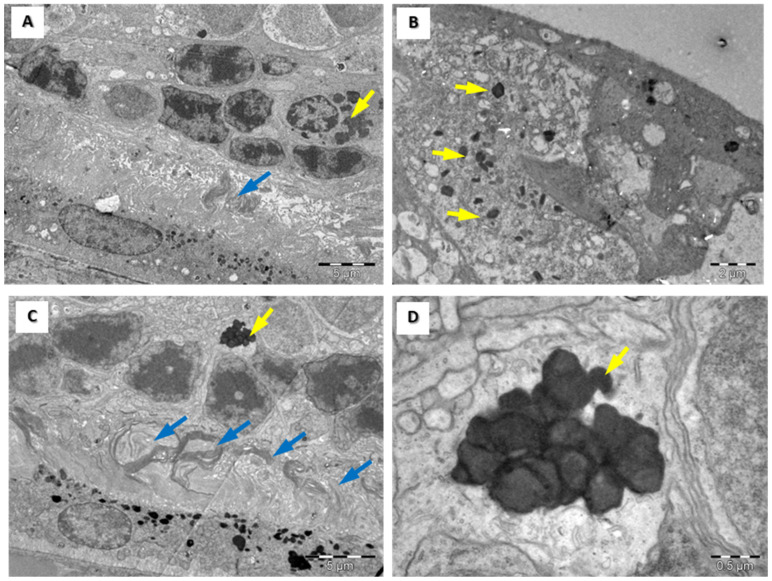
Transmission electron microscopy images of rat retinas from groups 3 A (**A**,**B**) and 3 B (**C**,**D**). Focal alteration (blue arrows) of rod and cone layer in group 3 A (**A**) and extensive number of lysosomes in rod and cone layer in group 3B (**D**). Increased number of lysosomes (yellow arrows) in groups 3 A and 3 B in the outer retinal layer (**A**,**C**) and inner retinal layer (**B**). Higher magnification from (**C**) of a lysosome in a Müller cell. Scale bars: 5 µm (**A**,**C**), 2 µm (**B**), and 0.5 µm (**D**).

In the inner nuclear layer, we observed horizontal, bipolar, amacrine, and Müller cells. Ganglion cells can be detected based on their bulky euchromatic nuclei, as well as a moderate number of cellular organelles (Figure 3G). A microscopic examination of retinas in subgroup 2 B revealed cytoplasmic bodies in the cone cells of the outer nuclear layer, in the inner nuclear layer cells, and in the ganglion cells (Figure 3C,F,I), as well as edema in the retinal ganglion cells. An ultrastructural examination of rat retinas in subgroup 3 A highlights subtle deterioration in the external segment of the photoreceptors, lysosomes in the inner nuclear layer cells, and ganglion cells (Figure 3B,E,H), as well as a few areas of focal alteration of the rod and cone layer (Figure 4A) and a few lysosomes in the outer retinal layer and inner retinal layer (Figure 4B). An ultrastructural examination of retinas in subgroup 3 B showed significant focal changes within the external segment of the photoreceptors and apoptotic cells among the cells of the inner nuclear layer, with an increase in chromatin density and nuclear fragmentation; we also observed bulky ganglion cells with multiple cellular lysosomal inclusions in the cells (Figure 4C,D).

**Figure 5:**
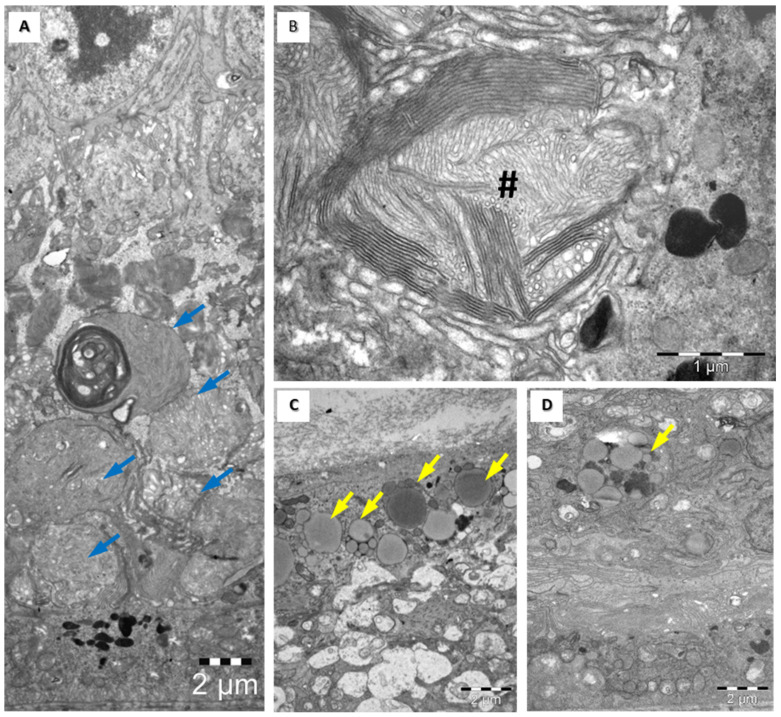
Transmission electron microscopy images of rat retinas from group 4 B underscore the significant impact of long-time administration of high doses of HCQ on the retinal structure. Highly unorganized rod and cone layer (**A**,**B**) and lipid droplet accumulations in the inner (**C**) and outer (**D**) layers of the retina. Rod/cone segments show aggregated membranes (blue arrows in (**A**))—scale bars 2 µm. Higher magnification of aggregated membranes shows their lamellar structures (**B**)—scale bars 1 µm.

An ultrastructural examination of rat retinas in subgroup 4 B highlighted significant changes in both the external and internal segments of the photoreceptor cells (cones), with an anarchic pattern (Figure 5A,B); rod/cone segments showing aggregated membranes (Figure 5A); lysosomes and lipid vacuoles in cells within the inner nuclear layer (bipolar cells and horizontal cells) (Figure 5C); and increased edematous retinal ganglion cell volume, with multiple dense cellular inclusions and lipid vacuoles in the outer layers of the retina (Figure 5D).

## 4. Discussion

The morphological key to the retinal toxicity induced by HCQ administration is the intracellular accumulation of lysosomes found in retinal ganglion cells and, apparently, within the inner nuclear and photoreceptor layers in a lower amount; this finding was observed in the group that received a daily dose of 100 mg/kg for a longer period (4 months)—subgroup 4 B. These findings suggest that retinal tissue toxicity may be induced by an increased dosage administered over a longer period.

A direct proportional relationship between the length of therapy and retinal ultrastructural-level effects in rats has been emphasized by Dunker et al. [13] and Yoshida et al. [14], but the analyzed drug in those experiments was chloroquine. Rats were treated with different doses of Chloroquine and retinal changes were analyzed after 8 and 12 weeks. After 8 weeks, lipidosis-like inclusions were found in the rat retinas. After 12 weeks, there was receptor cell degeneration and there were macrophage-like cells in the peripheral and central retina; all these changes induced more extreme deformation of the retina [13].

The exact mechanism of retinal toxicity remains uncertain. Previous studies have shown changes in outer retinal layers (the retinal pigment epithelium layer and photoreceptors) [15], while other researchers suspect damage to the structural level of retinal ganglion cells, the inner plexiform layer, or the retinal nerve fiber layer [6,7,8,9,10,11,12,13,14,15,16].

Lysosomes have been detected in previous studies on pigmented and albino rats that underwent chronic systemic exposure to chloroquine [17]. These cellular inclusion structures are like the cytosomes described by Johannessen: concentrically arranged membranous layers [18,19] detected in neuronal disorders with lysosomal deposits and cases of chloroquine retinopathy. This suggests that this drug induces a generalized alteration of lysosomal enzymes.

In a study published in 2004, Mahon et al. highlighted the effect of long exposure to chloroquine on male hooded Lister rats. The study emphasized that prolonged exposure to chloroquine leads to severe retinopathy that cannot be distinguished from pigmentary retinopathy, which is characterized by outer retina neurodegeneration [20]. The mechanism of retinal cell dysfunction is yet to be clarified. One hypothesis is that this effect could be explained by the existence of a continuous drug reservoir or the gradual decompensation of damaged cells during HCQ exposure [21].

## 5. Conclusions

Our in vivo research (optical and transmission electronic microscopy) captured histopathological and ultrastructural changes induced by chronic HCQ administration, using dose management at different periods, compared with a control group. Both microscopic assessments (OM and EM) highlighted a statistically significantly positive correlation between the long period of high-dose exposure and the destructive effect of the substance, indicating the retinal toxicity of chronic HCQ exposure. At both doses (50 mg/kg; 100 mg/kg) for longer administration periods, trophic preservation of the retinal pigment epithelium was observed.

If the chronic administration of this drug is associated with thickening in the retinal pigment epithelium, this could call into question HCQ’s protective effect on the macula. Although this speculation was contradicted by our electronic microscopy analysis, ultrastructural changes were observed in the third month of exposure to a single daily HCQ dose of 50 mg/kg; in this experimental group, we found subtle changes even in the photoreceptor cells, showing that the drug caused a cumulative retinal toxic effect through the generalized alteration of lysosomal enzymes.

Our research may be a step forward for future in vivo experimental studies, offering a detailed view of retinal changes induced by HCQ and a path to discovering a new therapeutic value for this substance, especially in disorders where local inflammation plays a role in the pathogenesis and progression of the disease.

## Figures and Tables

**Table 1:** Schematic presentation of groups and subgroups of experimental rats.

Treatment Duration (Months)	HCQ 50 mg/Kg (Group A) Total = 24 Rats	HCQ 100 mg/Kg (Group B) Total = 24 Rats	HCQ 200 mg/Kg (Group C) Total = 24 Rats	Saline 0.4 mL (Control Group) Total = 24 Rats
**1**	**1 A** (n = 6)	**1 B** (n = 6)	**1 C** (n = 6)	**1 M** (n = 6)
**2**	**2 A** (n = 6)	**2 B** (n = 6)	**2 C** (n = 6)	**2 M** (n = 6)
**3**	**3 A** (n = 6)	**3 B** (n = 6)	**3 C** (n = 6)	**3 M** (n = 6)
**4**	**4 A** (n = 6)	**4 B** (n = 6)	**4 C** (n = 6)	**4 M** (n = 6)

**Table 2:** Protocol for experimental rat procedures throughout the study.

Total: 96 RATS	Group A (50 mg/kg)	Group B (100 mg/kg)	Group C (200 mg/kg)	Control Group M (Saline 0.4 mL)
**Rats sacrificed every month (1, 2, 3, 4)**	6	6	-	6
**Deceased rats (spontaneously) within 48 h of the experiment**	0	0	24	0

**Table 3:** Mean values with standard deviations (SDs) of parameters followed in the study: * *p* < 0.01 vs. control, highly statistically significant; ** *p* < 0.05 vs. control, statistically significant; and *** *p* < 0.1 vs. control not statistically significant.

Groups	AVERAGES			
	Total Retinal Thickness (µm) ± SD	Inner Retinal Thickness (µm) ± SD	Outer Retinal Thickness (µm) ± SD	Retinal Pigment Epithelium Thickness (µm) ± SD
**Control group 1 M**	150.00 ± 1.10	88.00 ± 1.26	62.00 ± 1.10	13.50 ± 1.05
**Subgroup 1 A**	151.00 ± 0.89 ***	88.67 ± 1.03	62.33 ± 1.21	13.67 ± 0.82
**Subgroup 1 B**	149.83 ± 1.33	87.83 ± 1.33	62.00 ± 1.10	13.50 ± 1.05
**Control group 2 M**	150.00 ± 1.10	87.00 ± 0.89	64.00 ± 0.89	13.00 ± 1.41
**Subgroup 2 A**	154.00 ± 0.63 **	91.00 ± 1.10 *	63.00 ± 1.10 **	13.50 ± 0.82
**Subgroup 2 B**	137.50 ± 1.76 *	75.83 ± 0.75 *	61.67 ± 1.97 **	11.17 ± 1.17 **
**Control group 3 M**	150.00 ± 1.10	88.00 ± 1.26	62.00 ± 2.00	13.00 ± 0.89
**Subgroup 3 A**	157.50 ± 1.05 *	93.17 ± 0.75 *	64.33 ± 1.03 **	15.17 ± 1.17 *
**Subgroup 3 B**	133.80 ± 0.75 *	74.67 ± 0.82 *	59.17 ± 1.17 *	9.50 ± 0.55 *
**Control group 4 M**	151.00 ± 1.79	87.00 ± 0.84	64.00 ± 1.79	13.00 ± 1.55 *
**Subgroup 4 A**	162.00 ± 0.63 *	95.00 ± 0.89 *	67.00 ± 1.26 *	17.17 ± 0.75 *
**Subgroup 4 B**	115.00 ± 0.63 *	55.17 ± 0.98 *	59.83 ± 0.98 *	9.00 ± 0.89 *

## Data Availability

The original contributions presented in the study are included in the article material, further inquiries can be directed to the corresponding author.

## References

[1] Geamănu Pancă A., Popa-Cherecheanu A., Marinescu B., Geamănu C.D., Voinea L.M. (2014). Retinal Toxicity Associated with Chronic Exposure to Hydroxychloroquine and Its Ocular Screening. Review. J. Med. Life.

[2] Yam J.C.S., Kwok A.K.H. (2006). Ocular Toxicity of Hydroxychloroquine. Hong Kong Med. J..

[3] Geamanu A., Editura Universitara “Carol Davila” (2020). Modificari Structurale Retiniene Datorate Efectului Expunerii La Hidroclorochina.

[4] Yahalomi T., Pikkel Y., Arnon R., Porat D., Pikkel J. (2023). The HIT Study-The Hydroxychloroquine Effect in the Treatment of Patients with Age-Related Macular Degeneration: A Randomized Controlled Trial. Medicina.

[5] Anderson D.H., Mullins R.F., Hageman G.S., Johnson L. (2002). V A Role for Local Inflammation in the Formation of Drusen in the Aging Eye. Am. J. Ophthalmol..

[6] Pasadhika S., Fishman G. (2010). Effects of chronic exposure to hydroxychloroquine or chloroquine on inner retinal structures. Eye.

[7] Hallberg A., Naeser P., Andersson A. (1990). Effects of long-term chloroquine exposure on the phospholipid metabolism in retina and pigment epithelium of the mouse. Acta Ophthalmol..

[8] in t’ Veld B.A., Ruitenberg A., Hofman A., Launer L.J., van Duijn C.M., Stijnen T., Breteler M.M., Stricker B.H. (2001). Nonsteroidal Antiinflammatory Drugs and the Risk of Alzheimer’s Disease. N. Engl. J. Med..

[9] McGeer P.L., Schulzer M., McGeer E.G. (1996). Arthritis and Anti-Inflammatory Agents as Possible Protective Factors for Alzheimer’s Disease: A Review of 17 Epidemiologic Studies. Neurology.

[10] McGeer P.L., Sibley J. (2005). Sparing of Age-Related Macular Degeneration in Rheumatoid Arthritis. Neurobiol. Aging.

[11] Melles R.B., Marmor M.F. (2022). Rapid Macular Thinning Is an Early Indicator of Hydroxychloroquine Retinal Toxicity. Ophthalmology.

[12] Manschot W.A., Lee W.R. (1984). Retinal neovascularisation arising from hyalinised blood vessels. Graefe’s Arch. Clin. Exp. Ophthalmol..

[13] Duncker G., Schmiederer M., Bredehorn T. (1995). Chloroquine-Induced Lipidosis in the Rat Retina: A Functional and Morphological Study. Ophthalmologica.

[14] Yoshida T., Fukatsu R., Tsuzuki K., Aizawa Y., Hayashi Y., Sasaki N., Takamaru Y., Fujii N., Takahata N. (1997). Amyloid Precursor Protein, A Beta and Amyloid-Associated Proteins Involved in Chloroquine Retinopathy in Rats--Immunopathological Studies. Brain Res..

[15] Duncker G., Bredehorn T. (1996). Chloroquine-Induced Lipidosis in the Rat Retina: Functional and Morphological Changes after Withdrawal of the Drug. Graefe’s Arch. Clin. Exp. Ophthalmol..

[16] Pasadhika S., Fishman G.A., Choi D., Shahidi M. (2010). Selective Thinning of the Perifoveal Inner Retina as an Early Sign of Hydroxychloroquine Retinal Toxicity. Eye.

[17] Abraham R., Hendy R.J. (1970). Irreversible Lysosomal Damage Induced by Chloroquine in the Retinae of Pigmented and Albino Rats. Exp. Mol. Pathol..

[18] Johannessen J.V. Cellular Pathology, Metabolic and Storage Diseases.

[19] Mondal K., Porter H., Cole J., Pandya H.K., Basu S.K., Khanam S., Chiu C.Y., Shah V., Stephenson D.J., Chalfant C.E. (2022). Hydroxychloroquine Causes Early Inner Retinal Toxicity and Affects Autophagosome-Lysosomal Pathway and Sphingolipid Metabolism in the Retina. Mol. Neurobiol..

[20] Mahon G.J., Anderson H.R., Gardiner T.A., McFarlane S., Archer D.B., Stitt A.W. (2004). Chloroquine causes lysosomal dysfunction in neural retina and RPE: Implications for retinopathy. Curr. Eye Res..

[21] Kader D.H.A., Sayed S.S.E., El-Ghamrawy T.A. (2007). Chloroquine-Induced Retinopathy in the Rat. Immunohistochemical and Ultrastructural Study. J. Med. Sci..

